# Impact of steroid hormone signals on *Drosophila *cell cycle during development

**DOI:** 10.1186/1747-1028-4-3

**Published:** 2009-01-20

**Authors:** Nicola Cranna, Leonie Quinn

**Affiliations:** 1 Department of Anatomy and Cell Biology, University of Melbourne, Parkville 3010, Melbourne, Australia

## Abstract

Metamorphosis of *Drosophila *involves proliferation, differentiation and death of larval tissues in order to form the adult fly. The major steroid hormone implicated in the larval-pupal transition and adult tissue modelling is ecdysone. Previous reviews have draw together studies connecting ecdysone signaling to the processes of apoptosis and differentiation. Here we discuss those reports connecting the ecdysone pulse to developmentally regulated cell cycle progression.

## Review

### The ecdysone steroid hormone controls insect development

The *Drosophila melanogaster *imaginal discs provide an ideal model for understanding how developmental signaling pathways control the cell proliferation required for animal growth and development. Imaginal discs develop into the adult head structures (eyes and antenna), appendages (wings and legs) and genitalia. The imaginal disc precursor cells arise early in embryonic development, where they are established and localised as groups of cells in specific regions of the embryo. Each imaginal disc develops from invaginations of the embryonic epithelium and by the early larval stage, consist of a ball of around 10–50 undifferentiated stem cells, which undergo massive growth and proliferation to comprise up to 100,000 cells by the end of the third larval instar. The imaginal discs start differentiation at the end of third instar and complete the process by the end of pupariation, when all adult structures such as the wings, legs and eyes have developed [1]. The third instar larval stage is therefore a critical stage of *Drosophila *development, containing the major growth and proliferation of all tissues required to form the adult fly [2]. Correct development of adult structures requires coordination of proliferation with the onset of cell differentiation in the imaginal discs.

#### EcR/USP structure and function

The major developmental hormone in *Drosophila*, the steroid hormone 20-hydroxyecdysone, commonly known as ecdysone, is secreted from the prothoracic gland (PG) and plays a major role in regulating imaginal disc development. Ecdysone release is controlled by a complex combination of upstream factors, including peptide hormones and neuropeptide signals [3]. For example, Prothoracicotropic hormone (PTTH) from the central nervous system (CNS) is required to regulate the synthesis and release of ecdysone from the PG [4]. Ecdysone pulses are required for all aspects of morphogenesis, starting with the formation of the body plan during late embryogenesis required to develop to first instar larvae and for the cuticle moulting at the end of the first and second instars. A large titre of ecdysone is released at the end of the third instar, in the wandering larvae in preparation for pupation, which marks the beginning of adult tissue metamorphosis [5-8]. The range of larval structures that respond to the ecdysone pulse at this transformation stage all elicit diverse cellular responses to achieve conversion from the larval tissue to the adult structures [9].

During metamorphosis, a cascade of gene transcription is triggered by ecdysone, which activates the ecdysone receptor (EcR), a member of the nuclear receptor family, and its receptor binding partner Ultraspiricle (USP) [5-8]. The *EcR *gene spans 77 kb in length, and through the use of two promoters and as a result of alternate splicing, encodes three major protein isoforms EcR-A, EcR-B1, EcR-B2. The EcR-A, EcR-B1, EcR-B2 isoforms have conserved DNA binding domains and ligand binding domains but differ in their N-terminal regions, with variable N terminal domains of 197, 226 and 17 amino acid residues, respectively [8,10]. Although EcR can bind ecdysone alone, optimal binding to the ecdysone response elements (*EcRE*) and activation of transcriptional targets requires the addition of USP [11,12].

USP exhibits a strong structural and functional similarity to the orthologous vertebrate retinoid × receptor (RXR) [13,14]. Like RXR, which forms heterodimers with non-steroid receptors for thyroid hormone, retinoic acid and vitamin D, and thereby activates them for DNA-binding [15], USP interacts with each of the EcR isoforms to form DNA-binding heterodimers [9,13]. *Drosophila *EcRs are therefore analogous to the vertebrate family of RXR heterodimeric receptors rather than the vertebrate family of steroid hormone receptors, which bind DNA as homodimers [16]. In the presence of the ecdysone ligand, the appropriate EcR nuclear receptor isoform dimerizes with Ultraspiricle (USP), and the complex is stabilised by the ecdysone ligand to allow efficient binding to the ecdysone response element (*EcRE*) [17,18] and transcriptional activation of ecdysone-responsive genes (such as *BR-C, E74, E75*) [19-23]. Early genes encode transcription factors that activate late genes and this hierarchy of gene activation is required for subsequent development [5]. To increase the output of the ecdysone pulse, EcR provides an autoregulatory loop by increasing the level of it's own transcription in order to further increase receptor levels in response to the ecdysone ligand [8].

#### The Ecdysone pulse drives cell death and differentiation

An essential process driven by the ecdysone pulse is the removal of larval tissues no longer required in the adult [24]. The process of steroid hormone driven apoptosis is an important part of tissue remodelling, whereby selective death removes unwanted cells towards generating the mature structure [25]. For example, the histolysis of the larval salivary gland and midgut at the end of metamorphosis is stage-specific, ecdysone triggered, programmed cell death, which results in the removal of the component of these larval structures no longer required in the adult fly. Consistent with this, previous studies have shown that cell death activators are upregulated in the third instar larval tissues, including the salivary glands and midgut in response to ecdysone (reviewed [24,26,27]).

The ecdysone is pulse is also essential for differentiation and patterning of the larval imaginal tissues required for development of adult structures [17,19,28,29]. As cell division and patterning are tightly linked in *Drosophila *imaginal tissues the process of metamorphosis controlled by ecdysone will therefore involve coordination of the developmental signals that regulate proliferation and differentiation. Although much work has focused on the downstream targets linking the ecdysone pathway to programmed cell death and cell differentiation [24,26,27], the relationship between ecdysone and cell cycle is less clear. Here we integrate evidence providing a link between the ecdysone pulse and cell cycle progression in *Drosophila*.

### Linking the Ecdysone pulse to cell cycle

#### Conservation of cell proliferation machinery in Drosophila

In *Drosophila*, cell growth and cell cycle progression are regulated by a number of key genes, which have been shown to control the cell cycle in an analogous manner in all multicellular organisms. These include the *Drosophila *orthologue of the mammalian *c-myc *transcription factor and oncogene, dMyc, which drives growth and progression through G1 to S-phase [30], the essential G1 to S-phase Cyclin complex, Cyclin E (CycE) and it's Cyclin-dependent-kinase (Cdk) partner Cdk2, which triggers S-phase by promoting DNA replication [31-33], and the *Drosophila *orthologue of the Cdc25 phosphatase, String (Stg), which is required for G2/M progression and promotes mitotic entry by activating the Cdk1/Cyclin B complex [34]. CycE and Stg are the rate limiting factors for S-phase and mitosis, respectively, and both are activated by the *Drosophila *orthologue of human E2F1 protein, dE2F1 [32]. dE2F1 responds to the relevant Cdk-Cyclin complex (CycE/Cdk2 for S-phase and CycB/Cdk1 for mitosis) to coordinate cell cycle progression from G1 to S-phase and G2 into mitosis [35].

During metamorphosis, growth of larval tissues occurs in an ecdysone-dependent manner to produce adult structures. For example, during pupal development the larval midgut is removed by apoptosis and is replaced through proliferation of imaginal tissues to form the adult midgut [26,36]. Microarray analysis has revealed that the ecdysone signal is associated with the activation of key cell cycle genes, including *Cyclin B, Cdc2 *and *Cyclin D*, during the initiation of midgut metamorphosis [37]. Analysis of *EcR *null mutants revealed that EcR function was necessary for the cell cycle and growth genes to be activated in the larval midgut, suggesting that the ecdysone pathway is required for cell division control.

#### Cell extrinsic effects of ecdysone on larval growth

The ecdysone pulse has been shown to act non-autonomously to affect larval growth. These cell extrinsic effects of the ecdysone pathway are reviewed elsewhere [38-41] and therefore only mentioned briefly here. This control of *Drosophila *larval growth and final body size occurs non-autonomously, at least in part through interactions between the ecdysone and insulin pathways. The insulin signaling pathway acts in the prothoracic gland (PG) to regulate the release of ecdysone, therefore influencing the rate and duration of larval growth [38,39,42-45]. For instance, increased PG growth occurs when PI3-kinase (PI3K, a downstream regulator of the insulin pathway) is upregulated in the PG [42,44]. The PG overgrowth causes accelerated metamorphosis, which results in reduced adult size due to the rapid progression through the larval growth stage. Precocious ecdysone release, as measured by premature increase in levels of the early response ecdysone genes, correlates with this disruption to larval growth. Conversely, reducing growth of the PG, using a dominant negative form of PI3K, resulted in a longer larval growth period and larger adults due to slower ecdysone release and delayed onset of pupariation. More recently it has been shown that Target of Rapamycin (TOR) may link the ecdysone regulated development to the PI3K mediated growth pathways [46] (reviewed [41]).

Correct timing of the critical peak in ecdysone is therefore essential for controlling larval growth and adult body size, but how does the ecdysone pulse achieve these changes in cell growth and cell cycle progression within the larval tissues? In particular, how does ecdysone connect with the major developmental signaling pathways that regulate cell cycle patterning in *Drosophila*?

### The ecdysone pathway mediates Morphogenetic furrow progression in the larval eye imaginal disc

**Figure 1:**
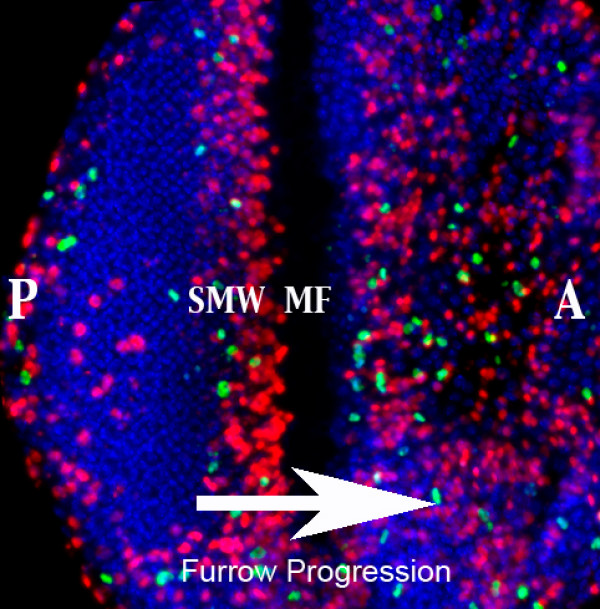
**Eye imaginal disc differentiation occurs in a wave that moves from posterior (P) to anterior (A)**. The margin between the asynchronously dividing anterior cells and the differentiated posterior cells is marked by the morphogenetic furrow (MF), where cells are delayed in G1. Mitotic division cycles become synchronized in the "Second Mitotic Wave" (SMW), which is composed of a tight band of DNA synthesis (marked by BrdU in red) and mitosis (marked by PH3 in green). The differentiated ommatidial clusters posterior of the furrow can be seen with the DNA stain in blue.

The *Drosophila *eye is composed of a highly organised array of photoreceptor clusters or ommatidia, which develop from an epithelial monolayer known as the eye imaginal disc (Figure 1). Differentiation of the ommatidia occurs in a wave that moves from the posterior toward the anterior [47]. The margin between the asynchronously dividing anterior cells and the differentiated posterior cells is marked by the morphogenetic furrow (MF) [48]. Mitotic division cycles become synchronized in the MF where cells are delayed in G1 and a subset of photoreceptor cells are specified. The remaining retinal cells synchronously re-enter the cell cycle in the "Second Mitotic Wave" (SMW), which is composed of a tight band of DNA synthesis and mitosis (Figure 1). These final cell divisions provide the cells required for differentiation of the ommatidial structures that form the adult eye [48,49].

Coordination of proliferation and patterning in the eye imaginal disc depends on key signaling pathways, such as Wingless (Wg), Hedgehog (Hh), Decapentaplegic (Dpp) and Notch [50], which are conserved in vertebrates where they are critical mediators of development and disease [51]. Microarray analysis has linked the ecdysone pulse during metamorphosis to transcriptional changes in mitogenic signaling molecules (described in detail below), which are essential for coordinating cell cycle and patterning of imaginal tissues. The observation that ecdysone signaling was essential for the activation of factors involved in developmental signaling pathways such as Wg, Notch and Dpp, suggests there might be many connections between the ecdysone pulse, signaling pathways and cell cycle regulation during metamorphosis in *Drosophila *[37].

#### The Hedgehog (Hh) and Dpp pathways control cell division in the larval eye

*Drosophila *eye development is dependent on *hedgehog *(*hh*) expression posterior to the MF [52,53] and *decapentaplegic *(*dpp*) expression within the MF [54]. *Drosophila *Dpp is a member of the mammalian transforming growth factor-beta (TGF-beta) family of secreted proteins. TGF-beta can behave as a tumour-suppressor or oncogene depending on the tissue microenvironment, thus pathway inhibition or activation can result in cancer progression [55-61]. Aberrant Hh signaling has also been associated with numerous human cancers, with much literature linking activation of the pathway with increased tumour progression [62-66].

In the eye disc, the Hh and Dpp signaling pathways regulate key cell cycle genes to mediate cell cycle arrest or progression. Cell cycle arrest occurs in G1 phase within and anterior to the MF in response to Dpp [67,68]. Dpp and Hedgehog (Hh) act redundantly to ensure G1 arrest, thus cells unable to respond to Dpp will arrest later in response to Hh [69]. Dpp and Hh are both required to ensure Cyclin E and dE2F1 are inhibited and the cells comprising the MF undergo a coordinated G1 arrest [70]. In addition to the cell cycle inhibitory role of Hh in the anterior of the MF, Hh acts to promote cell division in the SMW by upregulating Cyclin D to promote cell growth and Cyclin E to drive S-phase entry [71]. These contrasting roles of Hh in different cells indicate that levels of Hh may be important to dictate cell cycle outcome or that other factors are involved. Indeed Notch signaling has been shown to be critical to drive S-phase entry in the SMW [72,73].

#### Ecdysone and developmental cell cycle control in the eye

Studies in the eye primordium of the moth, *Manduca sexta*, suggest that progression of the MF, including proliferation and differentiation of ommatidial clusters, requires ecdysone. Eye primordia proliferation responds to a critical concentration of ecdysone and below this threshold cells arrest in the G2 phase of the cell cycle [74]. Premature exposure to high levels of ecdysone will also result in MF arrest and precocious maturation of ommatida [75]. These cell cycle responses to ecdysone are consistent with the moderate ecdysone pulse during the larval stage stimulating eye proliferation, whilst the high levels of ecdysone released after pupariation would be required for cell cycle exit and eye maturation.

The ecdysone pathway has also been implicated in regulation of MF progression in the *Drosophila *larval eye imaginal disc [76]. The *ecdysoneless *mutation (*ecd-ts*) is a hypomorphic temperature-sensitive allele, which reduces ecdysone secretion from the ring gland [77]. Homozygous *ecd-ts *flies show eye defects when shifted to the restrictive temperature during the third instar larval stage [76]. Consistent with the MF moving much more slowly than normal in the *ecd-ts *mutant, delayed eye differentiation was shown using the Elav marker. This phenotype is similar to phenotypes resulting from *hh *loss of function [53]. Consistent with *hh *being a downstream target of the ecdysone signal, decreased levels of Hh protein were detected posterior to the MF in *ecd-ts *larval eye discs [76]. Delayed MF progression would be consistent with a requirement for Hh in activation of the S-phase genes Cyclin D and Cyclin E and therefore cell cycle re-entry in the SMW [71]. The failure of MF movement in *ecd-ts *mutants was attributed to impaired cell cycle progression as S-phase numbers were dramatically decreased in the SMW [76]. Consistent with reduction of cell division within the SMW, levels of the mitotic cyclin, Cyclin B, were also reduced posterior to the MF [76].

USP also regulates cell cycle and differentiation in developing larval imaginal discs. Loss-of-function *usp *clones spanning the morphogenetic furrow in larval eye imaginal discs show an anterior shift in expression of the MF-specific marker Dpp, consistent with premature progression of the MF and a role for USP in repressing morphogenetic furrow movement [78]. Loss of functional USP affects many genes involved in cell fate specification in the eye, including the differentiation markers Spalt and Atonal [78]. Although expression of these differentiation markers occurs prematurely, specification of cells contributing to the ommatidia occurs normally. The cell cycle analysis of *usp *mutant clones suggested that although the MF was advanced, cell cycle progression was disrupted in the SMW. First staining for Cyclin A, as a marker for cells in either S or G2 phase, revealed fewer Cyclin A-positive cells in *usp*- clones posterior to the morphogenetic furrow [79]. Similarly, although the Cyclin B band was not shifted in *usp- *clones posterior to the MF, the numbers of cells expressing Cyclin B were reduced [80]. The reduction in cell cycle markers posterior of the MF suggests that USP is required for cell cycle progression in the SMW. In support of cell cycle induction in the SMW depending on the presence of USP protein, *usp *overexpression using the *GMR-*promoter, which is only expressed posterior of the furrow, can rescue the loss of Cyclin B in the *usp *mutant clone. As progression through the SMW and differentiation are tightly coupled, the reduced cell cycles in *usp*-/- clones may be associated with the premature differentiation observed [78].

Thus reduction in either ecdysone or USP results in reduced cell cycles, yet *usp *mutations increase the rate of MF movement [78-80] and loss of ecdysone stops the MF [76,81]. One explanation for these observations is that in the absence of ligand, the EcR/USP heterodimer normally acts as a repressor at certain *EcRE*s. For these target genes ecdysone would be required to relieve the transcriptional repression caused by unliganded binding of the EcR/USP complex. This idea emerged from the finding that the *Broad-complex *(*BR-C*), which encodes a family of zinc-finger transcription factors upregulated in response to high ecdysone titres [82], becomes ectopically expressed in wing imaginal disc cells loss-of-function for either *usp *[83] or *EcR *[84]. Although concrete evidence is lacking, the idea is that the early (pre-ecdysone pulse) repressive effect of the EcR/USP heterodimer at the *BR-C *promoter will be lost in either *EcR *or *usp *mutants.

The apparently contradictory effects of USP and ecdysone in the eye might therefore be a consequence of the differential effects of the pathway on *BR-C *transcription. The Z1 isoform of the *BR-C *(*BrC-Z1*) is normally expressed posterior to the MF but not anterior to the MF [85,86] and reduced induction of *BrC-Z1 *occurs in *ecd-ts *eye discs [76]. Loss of USP function has the opposite effect, leading to high level BrC-Z1 protein expression both anterior and posterior to the MF, which might occur as a consequence of de-repression of *BR-C *transcription [81]. This high level of BrC-Z1 protein in *usp *mutant clones may explain the MF advancement phenotypes, as ectopic BrC-Z1 protein has been shown to induce premature differentiation of photoreceptor cells [78-80].

Yet even though *BrC-Z1 *expression is downregulated in *ecd-ts *mutants [76], *BrC-Z1 *loss of functioneye imaginal discs are phenotypically different [79], suggesting that other downstream transcriptional targets of the ecdysone pathway mediate the reported effects on eye development. Like *ecd-ts*, impaired *BrC-Z1 *function results in decreased levels of Hh, defective MF progression and photoreceptor recruitment. However, unlike the findings for *ecd-ts*, reduced levels of Cyclin B were not detected in *BrC-Z1 *loss of function clones [79]. Rather loss of *BrC-Z1 *function results in defects in ommatidial assembly, suggesting a role for *BR-C *in post-MF differentiation rather than cell cycle regulation in the SMW [81]. This suggests that some ecdysone regulation in the eye is mediated by BrC-Z1, but that an alternate target(s) of the ecdysone pathway regulates the cell cycle activity required for cell cycles in the second mitotic wave.

#### BR-C regulates endocycles in the ovary

Although a direct cell cycle role has not been demonstrated in the eye, the ecdysone-responsive *BR-C *has been implicated in regulating DNA synthesis in the adult ovary during oogenesis. Ectopic *BR-C *expression leads to ectopic G1 to S-phase endoreplication cycles during oogenesis, consistent with the ecdysone pathway promoting DNA replication [87]. These studies suggest ecdysone is required for endocycles, which are cycles of DNA replication (S-phase progression) without cell division required to amplify specific regions of the genome (the *chorion *genes) required for formation of the egg shell [reviewed in [88]. *BR-C *loss of function causes premature arrest of *chorion *gene amplification, whereas overexpression of *BR-C *isoforms lead to the formation of additional foci of BrdU incorporation in follicle cells [89]. *BR-C *most likely promotes endoreplication in the *Drosophila *ovary via the key cell growth and S-phase regulators, dMyc [90] and Cyclin E [31,91,92].

### The ecdysone pathway regulates cell cycle progression in the larval wing disc

#### Developmental patterning of wing disc cell cycles

**Figure 2:**
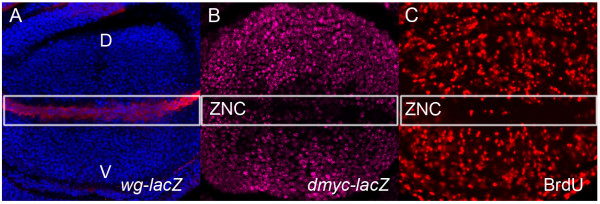
**Wg protein, *dmyc *expression and cell cycle patterning in the *Drosophila *wing pouch**. (A) Wg protein (red) is strongly expressed along the dorsal-ventral boundary of the wing pouch. (B) α-gal antibody staining (pink) of *dmyc-lacZ *(*w*^67*c*23^*P{lacW}l(1)G0354*^*G*0354^*; *[109]) discs shows a pattern consistent with *dmyc *transcription throughout the cycling cells of the pouch and downregulation of *dmyc *within the G1 arrested cells of the ZNC. (C) The zone of non-proliferating cells (ZNC) can be seen by the reduced BrdU staining (red) for S-phase along the dorsal-ventral boundary. Wing imaginal discs are aligned dorsal (D) to the top of the image, ventral (V) the bottom.

The larval wing disc is also comprised of an epithelial sheet, which can be divided into distinct domains based on cell fate in the adult wing; the notum, hinge and pouch. The wing pouch, which ultimately forms the adult wing blade, has been a focus for studying signals impacting upon the cell cycle. The morphogenesis of the wing blade involves patterned cell cycles that are tightly regulated and the signaling pathways affecting these cell cycles are well characterised [30,93-96]. A key signaling molecule in the morphogenesis of the wing is the Wingless (Wg) protein, a member of the Wnt family of secreted morphogens. Wg is secreted in a band across the dorsal-ventral (D/V) boundary in the wing pouch Figure 2[97] and is essential for cell cycle arrest in a region of the wing disc called the "Zone of Non-Proliferating Cells", or ZNC, at the end of larval development. The Wg pathway acts to downregulate the key cell cycle genes; including *dmyc*, *cycE*, *dE2F1 *and *stg*, to link the Wg patterning signal to proliferation [30,94,95,98]. The cell cycle arrest in the ZNC mediated by Wg is required for these cells to differentiate and develop into the adult wing blade (Figure 2) [30,94].

Cross-talk between the Wg pathway and other signaling pathways is required to coordinate proliferation and patterning of the wing imaginal disc. Dpp is expressed in a band of cells in the anterior compartment along the anterior-posterior boundary [99] and is required for cell cycle progression and tissue growth [100]. Proliferation is dependent on careful regulation of the relative levels of the DPP and Wg signaling pathways [reviewed in [101]. The Hedgehog (Hh) [102] and Notch (N) [103] pathways are key upstream regulators of Wg in the wing disc. Notch activity also plays a role in cell cycle arrest during wing development [94,104]. Notch is activated in cells along the dorso-ventral (D/V) boundary (ZNC) of the wing disc, where it is required for Wg expression [103]. The activation of Wg target genes *achaete *(*ac*) and *scute *(*sc*) specifically within the anterior compartment of the cells flanking the D/V boundary results in downregulation of the mitotic inducer, Cdc25c/Stg, to arrest these cells in G2 [94]. The expression of Notch specifically within the D/V boundary prevents the G2 arrest, allowing Wg to mediate G1 arrest within the anterior cells comprising the D/V boundary and all cells comprising the posterior compartment ZNC (Figure 2) [30,94]. The interplay of these signaling pathways therefore achieves the pattern of cell cycles within the wing pouch required for wing disc differentiation.

#### Ecdysone regulates the transcription factor Crol which acts to repress wg transcription to control wing disc cell cycles

**Figure 3:**
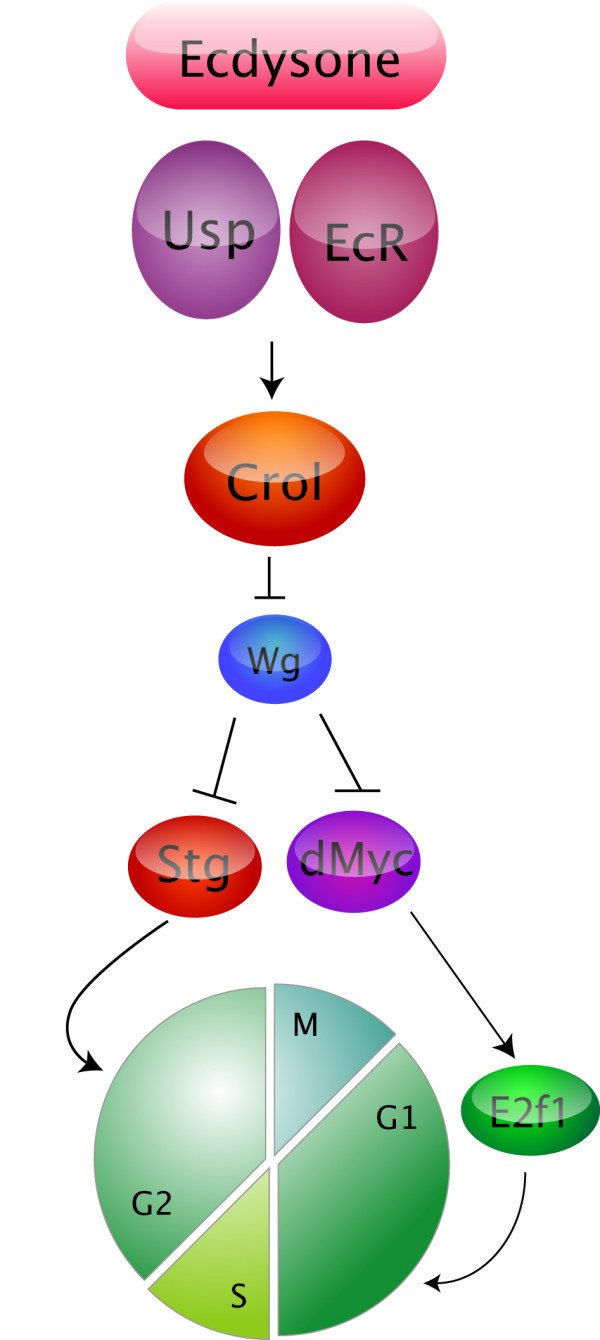
**Working model connecting Crol to steroid hormone signaling and cell cycle progression in the wing pouch**. Crol is up-regulated in response to ecdysone signaling and increased Crol results in decreased *wg *mRNA expression. Reduced Wg signaling leads to increased *dmyc *expression to drive S-phase and mitosis via increased Stg.

Our recent work revealed a link between the ecdysone pulse, the Wg pathway and developmental cell cycle regulation in the wing [105]. Previous studies have shown that Crol, which is a zinc finger transcription factor, is activated in late larval imaginal discs by the steroid hormone ecdysone [19]. Pupal lethal, hypomorphic *crol *mutants (*crol*^4418^) have defects in ecdysone-induced gene expression [19]. We demonstrated that Crol is required for cell cycle progression in the wing imaginal disc [105]. First, *crol *mutant clones in the wing pouch fail to proliferate, whilst overexpression of *crol *results in ectopic proliferation in the larval wing disc ZNC, suggesting Crol is both necessary and sufficient for third instar larval wing cell cycles. Crol is also required to downregulate the Wg pathway, which normally acts in the ZNC to drive cell cycle exit and differentiation (Figure 2). Therefore, by inhibiting the Wg pathway, *crol *positively drives cell cycle and potentially provides a link between the ecdysone pathway and the developmental signals that regulate cell cycle (Figure 3).

#### The EcR pathway is required for cell cycle progression in the wing

To determine whether ecdysone signaling via the EcR normally plays a role in cell cycle regulation we used two independent dominant negative lines to inactivate signaling through the EcR/USP/ecdysone complex; first the *EcRA *dominant negative (dN) receptor (EcRAdN), which still binds ecdysone, USP and the *EcRE*, but is completely defective in the activation of target-gene transcription due to a mutation in the ligand binding domain (LBD) [106]; and second the EcR-B2 dominant negative receptor, which dimerizes with USP and binds the *EcRE*, but cannot bind ecdysone, thus preventing optimal activation of ecdysone responsive genes [106,107].

**Figure 4:**
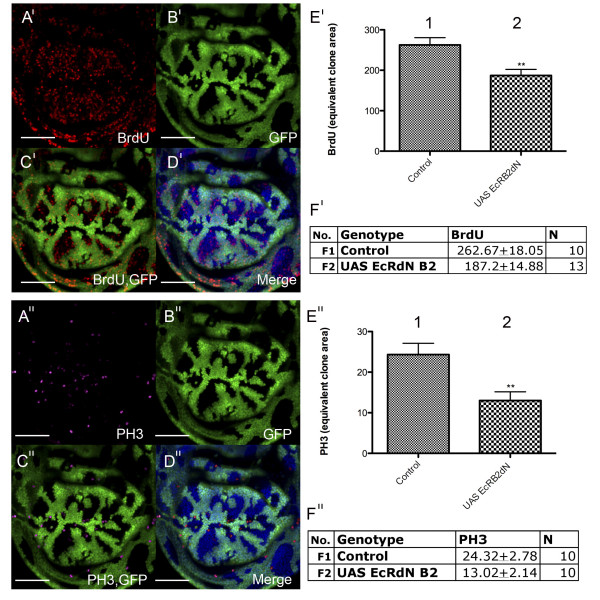
**EcR signaling is required for cell cycle progression in the wing disc**. (A'-D') Overexpression of *UAS-EcRBdN *in flip out clones [108] (A') BrdU (red) (B') GFP (green) positively marks clones (C') Overlay of GFP and BrdU (D') Merge of GFP and BrdU with DNA (DAPI) staining (blue) to show the nuclei of apical cells in the wing pouch. (E') Quantification of S-phase in clones overexpressing *UAS-EcRBdN *(E2'), and in an equivalent clone area of control tissue (E1'). A significant reduction (p = 0.0038) in S-phase was observed for *UAS-EcRBdN *compared to the control. (F1') Mean number of BrdU (S-phase) cells ± SEM for the control and (F2') for *UAS-EcRBdN*. (A"-D") Overexpression of *UAS-EcRBdN *(A") PH3 staining (purple) (B") GFP (green) positively marks clones (C") Overlay of GFP and PH3 (D") Merge of GFP and PH3 with DNA (DAPI) staining (blue). (E") Quantification of mitosis in clones overexpressing *UAS-EcRBdN *(E2"), and in an equivalent clone area of control tissue (E1"). A significant reduction (p = 0.0047) in the number of mitotic cells was observed for *UAS-EcRBdN *compared with the control. (F1") Mean number of PH3 cells ± SEM for the control and (F2") *UAS-EcRBdN*. Scale bars indicate 50 μm. N = sample size.

We have previously shown that blocking the EcR signal via overexpression of *EcRAdN *in third instar wing imaginal disc flip-out clones [108] results in cell cycle inhibition [105]. These results suggested that the EcR pathway was required for the normal pattern of wing imaginal disc cell cycles. Here we show the result of blocking the pathway with *EcRBdN *in wing imaginal disc clones (Figure 4A'–D'). *EcRBdN *overexpression results in wing disc clones with an overall decrease in BrdU positive cells. Quantification of BrdU revealed a significant decrease in S-phase progression in the *UAS-EcRBdN *clones compared with control clones (p = 0.0038, Figure 4E'–F'). Consistent with signaling through the EcR also being required to positively regulate progression through mitosis, clonal tissue also exhibited reduced numbers of PH3 positive cells (Figure 4A"–D") compared with control (p = 0.0047, Figure 4 E"–F"). Cell cycle analysis was carried out as described previously [105].

Therefore, blocking ecdysone pathway signaling using *UAS-EcR *dominant negative transgenes significantly reduces the number of cells progressing through the cell cycle. As both the *UAS-EcRAdN *and *UAS-EcRBdN *block the activation of ecdysone responsive genes, these findings suggest that targets of the ecdysone pathway are required for cell cycle progression in the *Drosophila *wing imaginal disc.

#### EcR signaling is required for dmyc transcription

**Figure 5:**
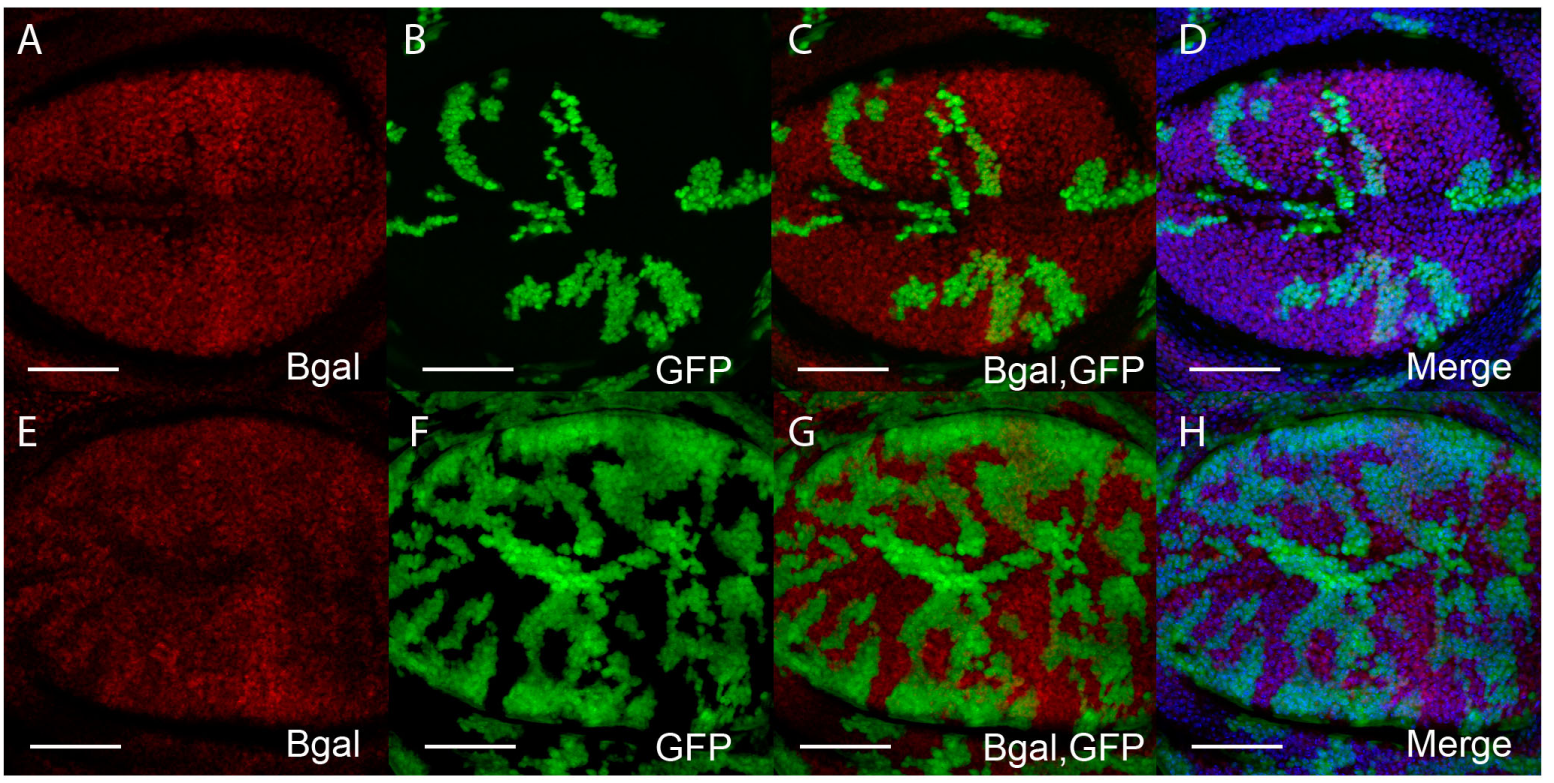
**EcR signaling is required for *dmyc *expression in wing imaginal discs**. (A-D) Discs with control clones to show *dmyc-lacZ *(*w*^67*c*23^*P{lacW}l(1)G0354*^*G*0354^*; *[109]) enhancer trap activity throughout the cycling cells of the wing pouch, with reduced β-gal antibody staining within the G1 arrested cells of the ZNC (A) β-gal staining (red) (B) GFP (green) positively marks clones (C) Overlay of GFP and β-gal, (D) Merge of GFP, β-gal with DNA (blue) to show the nuclei of apical cells in the wing pouch. (E-H) *UAS-EcRAdN *clones in the *dmyc-lacZ *background. (E) β-gal staining (red) (F) GFP (green) marked *UAS-EcRAdN *clones (G) Overlay of GFP and β-gal. (H) Merged image of GFP, β-gal and DNA (blue).

dMyc is a key mediator of growth and S-phase progression in the wing imaginal disc [30]. To test for changes to *dmyc *transcription *UAS-EcRAdN *flip-out clones were generated in the *dmyc-lacZ *enhancer trap background [109] (Figure 5). The control in Figure 5 (A–D) shows the expected pattern of *dmyc *transcription throughout the cycling cells of the wing pouch, with reduced staining within the cell cycle arrested cells of the ZNC [30]. Reduced β-gal staining in the GFP-positive *UAS-EcRAdN *clones (Figure 5 E–H), suggests that *dmyc *transcription is downregulated as a consequence of blocking EcR signaling.

These results are consistent with ecdysone signaling through EcR/USP normally being required for *dmyc *transcription. As increased dMyc leads to up-regulation of its cell cycle targets *cycE*, *cycD*, and *cdk4*, resulting in inactivation of *Rbf *and increased activity of the S-phase transcription factor E2f1 [98], this suggests EcR signaling might normally regulate S-phase progression by modulating *dmyc *levels (Figure 3).

#### EcR pathway is required for wg expresssion

**Figure 6:**
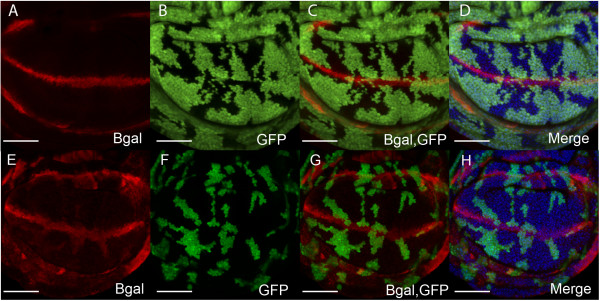
**Blocking EcR signaling upregulates *wg *transcription**. (A-D) *wg-lacZ *(*P{en1}wg*^*en*11^; [110]) activity in the wing pouch with control clonal tissue. (A) β-gal (red) (B) GFP (green) marked clonal tissue, (C) Overlay of GFP and β-gal, (D) Merged image of GFP, β-gal and DNA (blue). (E-H) *wg-lacZ *activity in pouch containing *UAS-EcRBdN *clones. (E) β-gal (red) (F) GFP (green) (G) Overlay of GFP and β-gal, (H) Merge of GFP, β-gal and DNA (blue). Scale bars indicate 50 μm.

We recently demonstrated that EcR signaling is required for repression of *wg *transcription. Consistent with the EcR pathway normally being required to repress *wg *transcription, expansion of the *wg *expression domain occurs in *UAS-EcRAdN *[105] and *UAS-EcRBdN *(Figure 6) flip-out clones generated in a *wg-lacZ *enhancer trap background [110]. These results suggest suppression of *wg *transcription in the wing pouch is dependent on the EcR pathway. Given that increased Wg protein causes reduction of cell cycle regulators such as *dmyc *and *stg*, leading to decreased cells in S-phase and mitosis in the pouch [30,94], this finding is consistent with the reduced cell cycles observed in *EcR *loss-of-function clones. Given that Crol is ecdysone responsive [19] and capable of repressing *wg *transcription [105], the proposed mechanism is that the ecdysone signal normally upregulates Crol to repress *wg *transcription and drive cell cycle progression in the pouch (Figure 3).

## Conclusion

The studies presented here show that the ecdysone pathway can modulate cell cycle progression in *Drosophila *by regulating mitogenic pathways. At the level of the whole animal, ecdysone controls larval growth and final body size through interactions with the insulin pathway [38-41]. During larval gut metamorphosis ecdysone activates cell cycle regulatory pathways such as Wg/Wnt, Notch and Dpp (TGFbeta) [37]. Within the larval eye imaginal disc ecdysone signaling is essential for cell cycle progression. The Hedgehog (Hh) pathway might be a downstream target of ecdysone posterior to the morphogenetic furrow (MF) [76], with the reduced Hh posterior to the MF in *ecd-ts *larval eye discs potentially leading to impaired S-phase gene activity and reduced cell cycle progression in the second mitotic wave (SMW) [71]. In addition to the cell cycle promoting role of Hh ahead of the MF, Hh acts in combination with Dpp to achieve a coordinated G1 arrest [69,70]. The shift in the Dpp band of expression in *usp- *clones, suggests the possibility that Dpp might be an ecdysone pathway target. Further work is required to understand how ecdysone might coordinate these developmental signals with the G1 arrest required for MF formation.

These studies strongly suggest a role for the ecdysone pathway and the USP receptor in furrow progression. However, previous analysis of *EcR *mutant clones led to the conclusion that EcR was not required for furrow progression [81]. This was surprising given the EcR isoforms are the major mediators of the ecdysone signal, combined with the *Maduca Sexta *[74,75] and *Drosophila *studies [76] that have demonstrated a clear requirement for ecdysone in MF progression. This lead the authors to propose a novel hormone transduction pathway involving an uncharacterized receptor to explain USP functioning independent of EcR in the eye. This could potentially occur via heterodimerisation of USP with one of the 16 orphan nuclear receptors identified in *Drosophila *[111]. In addition to it's partnership with EcR, USP has been found to heterodimerize with the orphan nuclear receptor, DHR38, to regulate cuticle formation [112,113]. The USP/DHR38 complex responds to a different class of ecdysteroids in larval fat body and epidermis in an EcR independent manner, which does not involve direct binding of the ecdysone ligand to either DHR38 or USP [114]. However, as *DHR38 *expression does not appear to be induced by ecdysteroids in the larval eye [114], it is unlikely that DHR38 partners USP during eye development. We believe it premature to rule out a function for EcR in MF progression as the absence of a furrow progression phenotype reported [81] may be a consequence of perdurance of EcR protein after clone induction. As studies using dominant negative EcR transgenes have shown that EcR is required for normal signaling and cell cycle progression in the wing (Figure 4, 5, 6[105]), before making conclusions about whether EcR is required for eye proliferation similar methods should be used to inhibit EcR activity.

In the wing imaginal disc, EcR activity and the ecdysone-responsive transcription factor Crol are required for cell cycle progression (Figure 4, 5)[105]. Crol affects the Wg pathway by downregulating *wg *transcription and driving cells through the Wg-mediated cell cycle arrest [105]. In support of ecdysone acting upstream of Crol to regulate the Wg pathway, blocking EcR activity in the wing results in increased *wg *transcription (Figure 6). As Wg is one of the key developmental signals required for inhibition of cell cycle progression in the wing pouch [30,94,95,98,115], this would be consistent with EcR regulating cell cycle by acting to increase levels of *crol *transcription, which will in turn decrease levels of Wg signaling (Figure 3). Thus we would predict that ecdysone/EcR/USP would normally act to upregulate Crol and drive cell cycle progression in the wing pouch.

As mentioned above a number of signaling pathways are required to coordinate cell division in the wing imaginal disc. The Hh pathway is critical for regulating *wg *transcription during wing development [102]. Ectopic Ci protein was not however detected in *crol *mutant clones, suggesting that Crol does not affect *wg *transcription indirectly via the Hh pathway [105]. Notch is required for Wg expression [103] and plays a critical role in cell cycle arrest during wing development [94,104]. The Notch target, En(spl)m7 was not however decreased in *crol *over-expressing cells, suggesting Notch signaling is not down-regulated by Crol [105]. The effects of Crol on cell cycle in the wing via down-regulation of *wg *transcription are therefore unlikely to be due to indirect effects on either the Notch or Hh pathways. Future studies are therefore aimed to determine whether Crol is a key downstream mediator of EcR signaling and whether it achieves repression of Wg by directly binding the *wg *promoter to down-regulate *wg *transcription.

## Competing interests

The authors declare that they have no competing interests.

## Authors' contributions

NC carried out experiments, data analysis and helped draft the manuscript. LQ conceived the study, carried out experiments, data analysis and drafted the manuscript. All authors read and approved the final manuscript.
